# A family history of *DUX4*: phylogenetic analysis of *DUXA*, *B*, *C *and *Duxbl *reveals the ancestral *DUX *gene

**DOI:** 10.1186/1471-2148-10-364

**Published:** 2010-11-26

**Authors:** Andreas Leidenroth, Jane E Hewitt

**Affiliations:** 1 Centre for Genetics and Genomics, School of Biology, The University of Nottingham, Queens Medical Centre, Nottingham NG7 2UH, UK

## Abstract

**Background:**

*DUX4 *is causally involved in the molecular pathogenesis of the neuromuscular disorder facioscapulohumeral muscular dystrophy (FSHD). It has previously been proposed to have arisen by retrotransposition of *DUXC*, one of four known intron-containing *DUX *genes. Here, we investigate the evolutionary history of this multi-member double-homeobox gene family in eutherian mammals.

**Results:**

Our analysis of the *DUX *family shows the distribution of different homologues across the mammalian class, including events of secondary loss. Phylogenetic comparison, analysis of gene structures and information from syntenic regions confirm the paralogous relationship of *Duxbl *and *DUXB *and characterize their relationship with *DUXA *and *DUXC*. We further identify *Duxbl *pseudogene orthologues in primates. A survey of non-mammalian genomes identified a single-homeobox gene (*sDUX*) as a likely representative homologue of the mammalian *DUX *ancestor before the homeobox duplication. Based on the gene structure maps, we suggest a possible mechanism for the generation of the *DUX *gene structure.

**Conclusions:**

Our study underlines how secondary loss of orthologues can obscure the true ancestry of individual gene family members. Their relationships should be considered when interpreting the relevance of functional data from *DUX4 *homologues such as *Dux *and *Duxbl *to FSHD.

## Background

Double homeobox genes are exclusive to placental mammals and characterized by two closely spaced homeoboxes of the PRD class[1]. The homeoboxes encode 60 amino acid homeodomains (HDs), an ancient, well studied DNA binding motif found in animals, plants and fungi in many transcription factors including the developmentally important *Hox *genes[2]. Our particular interest in the *DUX *family stems from the involvement of one member (*DUX4*) in the molecular pathogenesis of facioscapulohumeral muscular dystrophy (FSHD)[3]. In most cases of this genetic disorder, patients have a contraction of the 3.3 kb D4Z4 tandem repeat array on 4q35. When the genetic linkage between D4Z4 and FSHD was discovered, an intronless open reading frame (ORF) encoding a putative double-homeodomain protein was found to reside within each D4Z4 repeat unit[4] and named *DUX4*[5]. The *DUX4*-containing D4Z4 elements are present at high and variable copy number on 4q35 with 11 to >100 repeats in controls[6]. In FSHD, one allele is reduced in size to 1-10 repeats[6], which is accompanied by a change in chromatin packaging into a less repressive state[7-9]. A near identical tandem array is also present on 10q26, but contractions are not associated with FSHD.

D4Z4 contractions are only pathogenic if they occur on a particular haplotype background ("4qA161")[10,11]. It has been shown that the *DUX4 *ORF is discontinuously transcribed, with the ORF of the final repeat unit utilizing a polyadenylation signal in the pLAM region immediately distal to the D4Z4 array[12]. Recently, a landmark paper showed that only chromosomes of disease-permissive haplotypes such as 4qA161 carry this functional polyadenylation signal[13]. This study presents the strongest evidence yet for a *DUX4*-transcription mediated pathology in FSHD.

*DUX4 *related sequences are found in primates and Afrotheria and we previously showed that they have been conserved by selection[1]. In mouse, a related ORF known as *Dux *has been identified, which is also found in a tandem array organization[1]. The predicted Dux protein encoded within this array shares both homeodomains as well as a conserved C-terminal domain with DUX4[1]. In higher apes and humans, many hundreds of D4Z4 related sequences are dispersed throughout the genome, notably on the poorly assembled regions of the acrocentric chromosomes as well as the heterochromatic regions on 1q12[4,14]. In an interesting case study, the ongoing amplification of intronless *DUX *sequences has recently been characterized for a *DUX4 *homologue residing on the Y chromosome (*DUXY*)[15].

**Figure 1:**
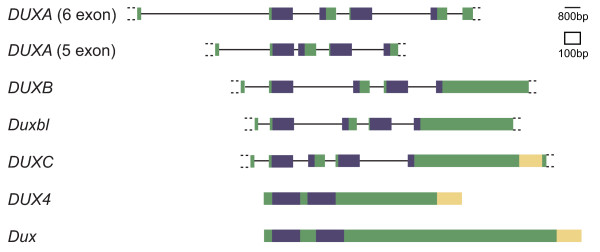
***DUX *family overview**. Representative structures of the four intron-containing and two intronless *DUX *genes are shown. Open reading frame in coloured boxes, homeobox sequences in dark blue. Conserved C-terminal domain of unknown function in yellow. Dotted boxes indicate UTR with uncertainty about transcriptional start and end sites. Note that introns and exons are drawn to different scales.

Interrogation of publicly available genome databases has resulted in the discovery of four intron-containing *DUX *genes (*A*, *B*, *C *and *Duxbl *= *BL*). *DUXA *and *DUXB *were originally identified in humans[16,17], but orthologues have also been found in a range of other mammals[1]. *DUXC *has been described only in armadillo, dog and cow[1]. Before the current study, *Duxbl *(named after *DUXB*-like) had been found only in mouse and rat genomes[1]. Independently, gene expression profiling experiments identified *Duxbl *as one of the genes upregulated in specific stages of mouse thymocyte development[18]. Figure 1 shows gene structures and conserved domains of the four intron-containing *DUX *homologues as well as the intronless *DUX4 *and rodent *Dux *sequences. The two homeoboxes in all four intron-containing genes are split across four exons, with splice junctions within the homeoboxes at equivalent positions.

The *in vivo *biological function of human *DUX4 *is still unknown, although a number of groups are addressing this question using experimental approaches based on exogenous expression of *DUX *genes[19-21]. Studies of *DUX *homologues may therefore contribute to our understanding of *DUX4 *function, but if such inferences are to be made with confidence, it is important that we understand how the genes and proteins studied relate to one another. Here, we comprehensively survey the distribution of intron-containing *DUX *genes in the mammalian class, characterize their gene structures and present data on their phylogenetic relationships. We also identify the putative ancestor of the *DUX *family and suggest a mutational mechanism that may have created the double homeobox gene structure.

## Results

### Cataloguing intact and inactive *DUX *homologues in placental mammals

**Figure 2:**
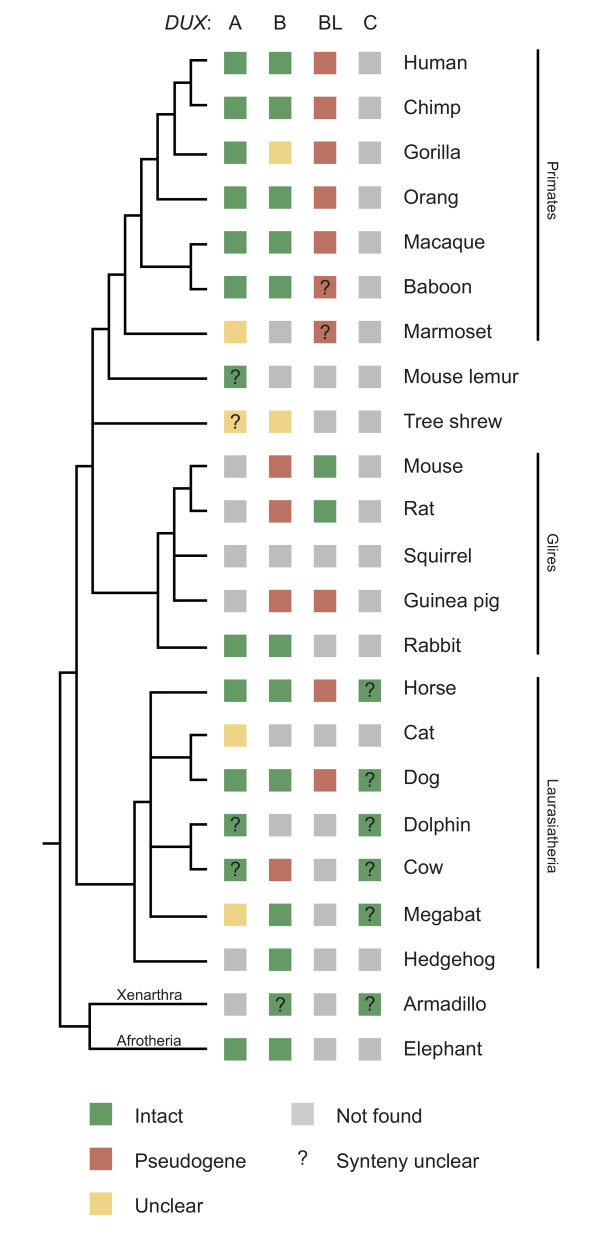
**Mammalian *DUX *distribution**. Summary of our mammalian *DUX *catalogue. Presumed functional genes (with intact ORF across all four homeobox exons, first exon may be unidentified) in green. *DUX *sequences with stop codons or deleted/missing exons in well sequenced regions in red. Putative *DUX *homologues with unclear functional status (mainly due to gaps in assembly) in yellow. Unless marked with ?, synteny was confirmed with anchor genes. Phylogenetic relationship between species according to references[28,44].

**Figure 3:**
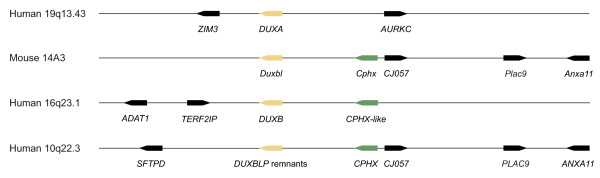
**Anchor genes**. Main anchor genes used to identify syntenic regions across species. *DUX *genes coloured yellow, anchor genes in black. *Cphx *as a marker of *Duxbl*/*DUXB *duplication shown in green. Gene sizes and intergenic spacing not drawn to scale. ENSEMBL access information: Human *Cphx *10q processed transcript (ENSG00000230091); Human *Cphx*-like 16q protein coding (ENSG00000232078); Mouse CJ057 (ENSG00000021867); Human *CJ057 *(ENSG00000133678).

We systematically searched all mammalian species in Figure 2 for each of the four known intron-containing *DUX *genes by tBLASTn. Searches were conducted on whole genome assemblies and separately on syntenic regions. The latter approach allowed the identification of decaying orthologues, whose degenerate amino acid sequences make them hard to identify in whole genome tBLASTn scans. Wherever possible, phylogenetic tree analysis of homeodomains, synteny information and gene structure data were combined to verify that an identified sequence represented the orthologue in that species. Figure 3 shows the relative locations of some of the neighbouring anchor genes used to establish synteny for *DUXA*, *B *and *BL*. For *DUXC*, no appropriate nearby anchors could be identified. *CJ057 *encodes a short, highly conserved protein that proved to be particularly useful due to its high conservation and linkage to *Duxbl *(Additional file 1). Figure 2 provides an overview of all *DUX *genes catalogued in this study.

### DUXA

*DUXA *orthologues can be found in most mammalian groups. Rabbits have an intact version, but we could not find any *DUXA *genes in other Glires. However, squirrel has an intronless *DUXA *sequence with an ORF spanning both homeoboxes, indicating that *DUXA *may either be present in an unsequenced region or have had its function maintained by a retrogene (such cases are not without precedent[22]).

*DUXA *was originally identified as a 6-exon gene in humans[16]. However, our survey of *DUXA *gene structures shows that the final exon seems to be exclusive to higher primates (Additional file 2). Mouse lemur, rabbit, Laurasiatheria and elephant share a stop codon at the equivalent position in exon 5, just before the location of the splice junction in primates. In dog, exon 5 is extended by 156bp and there is no GT splice donor signal at the position corresponding to that in primates (Additional file 3). Instead, there is an extended ORF, and the additional amino acids encoded by this putative exon share no similarity with the final primate exon. It is unlikely that Glires, Laurasiatheria and elephant have independently acquired a stop codon at the same position in exon 5. Instead, the ancestral *DUXA *probably contained five exons, with subsequent gain of a sixth exon in primates, effectively creating two *DUXA *subclasses. The dN/dS ratio of the DUXA C-terminal domain (after HD2) in a pairwise comparison of human, chimpanzee, macaque and orangutan shows an average across all pairs that is below 1 (0.27), indicating that the newly gained sequence has been maintained by purifying selection.

*DUXA *has spawned a large number of processed pseudogenes in primates as well as other species, indicating germline expression. Booth and Holland recently identified ten such pseudogenes in humans and reported a high number of EST matches for these sequences (*DUXAP1-10*)[23]. In a brief survey of human ESTs we found that some of these expressed sequences have less than 100% sequence identity with either *DUXA *or any of its known ten pseudogenes (data not shown). This indicates that yet more processed pseudogenes are likely to be dispersed throughout the genome, perhaps residing on the arms of acrocentric chromosomes (which have long been known to house intronless *DUX *sequences[4,14]). The presence of an EST sequence for *DUXA *in the databases has also been noted[16].

### DUXC

In addition to the previously reported copies of *DUXC *in armadillo, cow and dog[1], we have identified closely related homologues in horse, dolphin and megabat (Additional file 4). Interestingly, in all these species *DUXC *has undergone local duplications and is found in a tandem array organization. The dolphin assembly contains a contig with two intact *DUXC *copies and one pseudogene as well as the 5' end of a fourth copy, all arranged in tandem in the same orientation with a spacing of less than 5 kb between them. In megabat, one intact and one defective copy (containing a stop codon in HD2) are separated by a spacing of 25 kb. Trace archive sequence data from cow, dolphin, horse and dog also indicate a multi-copy, array-like organization (data not shown).

**Figure 4:**
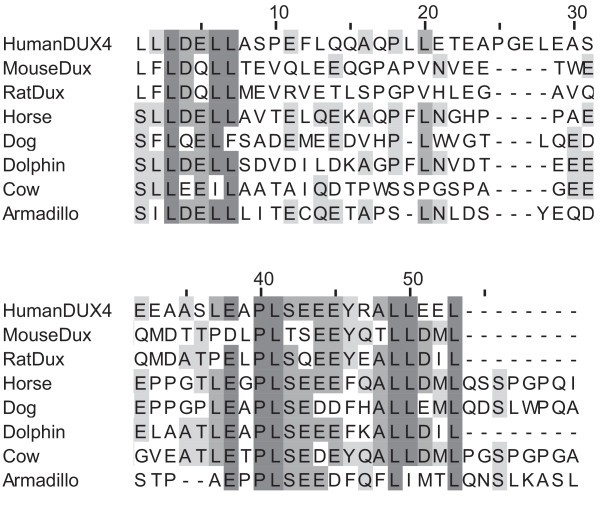
**Conserved C-terminal domain**. Amino acid alignment of conserved C-terminal domain found in DUXC, DUX4 and rodent Dux. All residues up to the stop codon are shown. Shading intensity indicates % of sequences that agree with consensus at that residue. Dark grey = >80%, grey = >60%, light grey = >40%, white = 40% or fewer. Figure produced with JalView 2.5.

The previously described conserved C-terminal domain of DUXC[1] is also found in the predicted protein of these newly-identified orthologues. Figure 4 shows an amino acid alignment demonstrating the conservation of this CTD not only between DUXC orthologues but also with human DUX4 and the predicted protein product of the rodent *Dux *arrays, evidence that they may be functionally related[1]. Maximum Likelihood analysis also clusters the two homeodomains of DUX4 with DUXC (Additional file 5 &6). Indeed, tBLASTn searches using DUXC as query return DUX4 sequences as top hits in primates. Thus far, we have not found any *DUXC *orthologues in primates and Glires, so it is possible that this homologue was lost in the common ancestor of the Euarchontoglires after the split from the Laurasiatheria. However, the lack of any suitable anchor genes for *DUXC *precludes analysis of homologous regions, and some orthologues may thus be missed.

### DUXB and Duxbl

Before this study the evolutionary relationship between *DUXB *and *Duxbl *was unclear, but our previous phylogenetic analysis suggested that they are not orthologues[1]. *Duxbl *had only been found in rodents[1,18], while *DUXB *sequences had been found in all major mammalian lineages except rodents[1]. Because genome-wide tBLASTn fails to detect decaying orthologues that have been secondarily lost and searches with low stringency pick up more distantly related homeobox genes instead (e.g. *MIX*), we used synteny information to identify genomic regions that might contain remnants of *DUXB *and *Duxbl *(Figure 3).

In mouse and rat, exons four and five of a *DUXB*-type gene are found next to the *TERF2IP *anchor, and each of these murine orthologues has a different stop codon mutation in exon five. In guinea pig and cow, exon four is absent but exons two, three and five are still present, again with different stop codon mutations in each species (Additional file 7).

**Figure 5:**
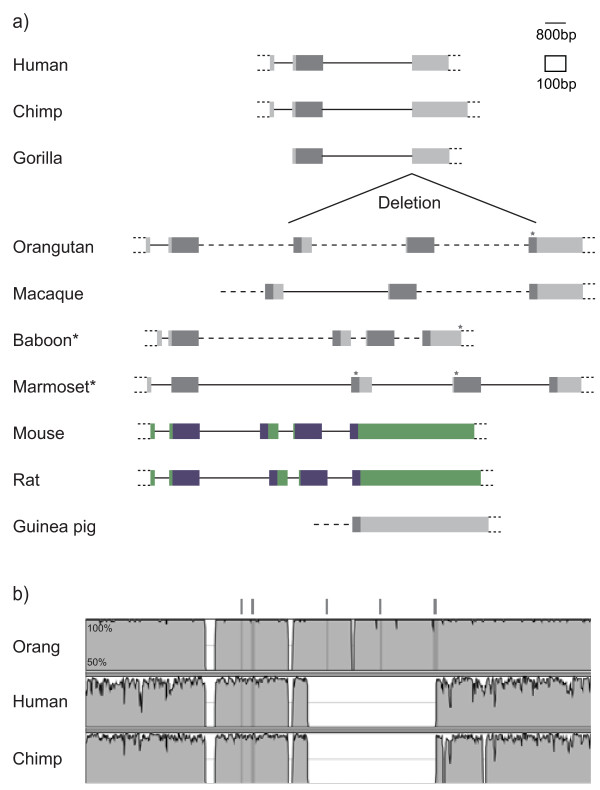
***Duxbl *genes and pseudogenes**. a) Overview of *Duxbl *pseudogenes and intact rodent *Duxbl*. Intact genes in colour, pseudogenes in grey. Dotted lines contain small gaps/contig holes in assembly. Asterisk next to species name indicates lack of synteny information. Star above exon = stop codon. b) Mapping of partial primate *Duxbl *deletion. Genomic nucleic acid alignment based on LAGAN in mVISTA of orangutan, human and chimpanzee *Duxbl *pseudogene loci. Conserved sequences shown as peaks, with *Duxbl *exons marked by dark bars. Gorilla was not included due to incomplete assembly.

For *Duxbl*, using *CJ057 *as an anchor, we found the final exon in guinea pig and decaying *Duxbl *pseudogenes containing various numbers of exons in primates (Figure 5a). In orangutan, baboon and marmoset, all five exons are present but have stop codons in positions unique to each species.

A recent 10 kb deletion occurred in the ape lineage, removing exons three, four and part of five in human, chimpanzee and gorilla (Figure 5b). It cannot be deduced whether *Duxbl *was still intact in these apes when the deletion occurred, or whether it was already a pseudogene, perhaps sharing the same stop codon as orangutan. However, the presence of intact (and presumably functional) *Duxbl *genes in mouse and rat and the relatively undisrupted structure of the primate pseudogenes in combination with independent inactivating mutations indicates that *Duxbl *was lost only recently in primates. The intact murine *Duxbl *also implies that the losses in primates and Laurasiatheria were independent events.

**Figure 6:**
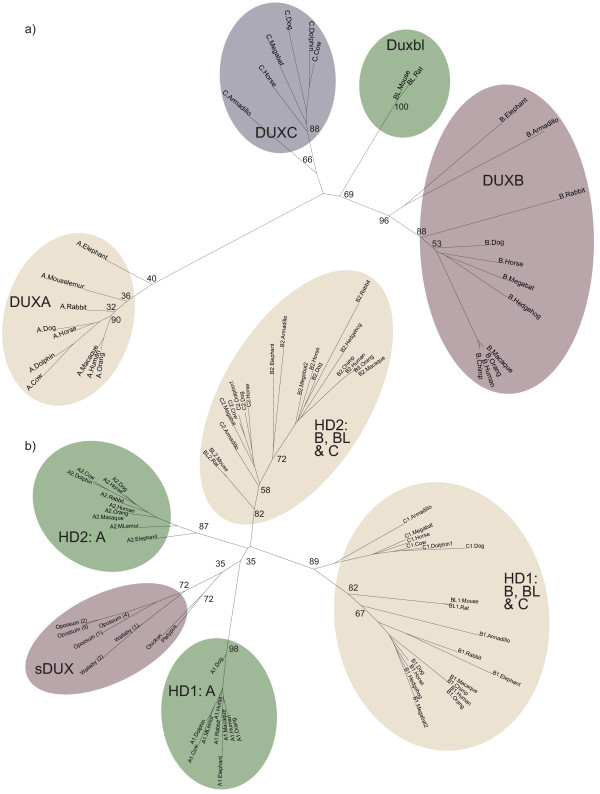
**Maximum Likelihood trees**. a) Tree based on concatenated homeodomain sequences (120 amino acids). b) Tree based on individual homeodomains (60 amino acids). Bootstrap values shown at internal nodes. Number after DUX name denotes homeodomain 1 or 2. Number in brackets indicates *sDUX *copy number.

Overall, these findings indicate a pattern of reciprocal loss and retention of *DUXB *and *Duxbl *in different lineages, although guinea pig is an exception, having lost both genes. The unrooted Maximum Likelihood tree in Figure 6 shows that Duxbl and DUXB cluster together, supporting a relatively recent divergence. We also noted the presence of a protein coding sequence (*Cphx*, itself encoding a homeodomain) adjacent to mouse *Duxbl*. *Cphx *has homologues next to both human *DUXB *and the human pseudogene orthologue of *Duxbl *(*DUXBLP*), indicative of a segmental duplication (Figure 3). This, taken together with the similar gene structure, high homeodomain identity and phylogenetic analysis, indicates that *DUXB *and *Duxbl *are paralogues that are probably more closely related to one another than either is to *DUXA *or *DUXC*.

*Duxbl *is also the *DUX *gene with the strongest experimental evidence for *in vivo *expression. *Duxbl *mRNA can readily be detected by RT-PCR in many mouse tissues including brain, liver, lung, kidney (unpublished data and references[18,24]), while protein expression has been shown in a subset of tissues[18,24].

### Identification of a single-homeobox gene linked to CJ057/ANXA11

**Figure 7:**
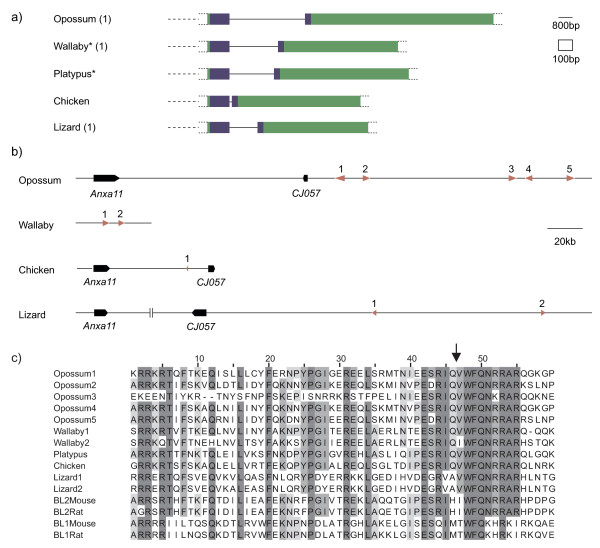
**Single-homeobox s*DUX***. a) Schematics of identified *sDUX *exons. Asterisk denotes missing synteny information. Number in brackets corresponds to copy number in diagram below. b) Map of *sDUX *genes relative to anchors. All locations, genes sizes and distances drawn to scale. Red arrows = two *sDUX *exons including intron. Orientation of arrow indicates strand orientation. Distance between lizard *Anxa11 *and *CJ057 *too large to show to scale. c) Amino acid alignment of homeodomain. Duxbl homeodomains 1 and 2 included for comparison. Arrow marks splice junction. Conserved residues shaded as in Figure 4.

In order to search for *DUX *homologues in more distantly related non-mammalian species, we extended our synteny-based analysis for *DUXA, B*, and *BL *to opossum, platypus and chicken. In these three species, the *DUXA *syntenic region could not be indentified with confidence, and we found no homeobox related sequences in the *DUXB *region (as defined by *ADAT1 *and *Terf2IP*). However, next to *CJ057 *orthologues, we found a putative *Duxbl*-related gene in both opossum and chicken, comprising at least two exons that encode a single homeodomain (Figure 7a). The position of the intron within the homeobox is at the equivalent location to that in *UXA*, *DUXB*, *Duxbl *and *DUXC *(Figure 7b). The large open reading frame in the second exon extends further than in *DUXB *and *Duxbl*. Close inspection of the local region did not identify any additional exons that could produce a double-homeobox gene.

Using the predicted ORF of this single-homeobox gene as a tBLASTn query, we also found homologues in platypus and wallaby (although lacking synteny information in either case), as well as anole lizard. Opossum, wallaby and lizard each have more than one copy of these two-exon pairs, in different orientations (Figure 7b). In both the wallaby and lizard assemblies, we found a retrotransposed copy containing both exons (joined at the homeobox splice junction). These retrocopies indicate that in the germline of these species at least, this sequence has been transcribed and spliced to encode a full homeobox. We suggest naming this single-homeobox *DUX*-related gene *sDUX *(for "single" homeodomain). Figure 7c shows an amino acid alignment of all sDUX homeodomains identified and includes rodent Duxbl homeodomains for comparison.

### Relationship of the intron-containing DUX genes

Despite extensive search efforts, no double-homeobox *DUX *genes have been identified in non-placental mammals here or previously[1]. Considering the similarity between the homeodomains of the four *DUX *homologues *A*, *B*, *BL *and *C *compared to other known homeodomain proteins[1] and their similar gene structures with identical splice positions in both homeoboxes, it is likely that the duplication of the *DUX *homeobox occurred only once in a single progenitor gene that gave rise to all four members of the *DUX *family. A Maximum Likelihood tree based not on concatenated but individual homeodomains supports this hypothesis (Figure 6b). For example, the first homeodomains of DUXB, Duxbl and DUXC all cluster together, while the second homeodomains of the same proteins form a separate cluster, suggesting that the two homeoboxes were already duplicated and divergent in the common ancestor of DUXB and DUXC. An analogous observation to such protein domain evolution among different homologues has been made on the level of gene evolution among different species[25]. When relationships between gene homologues (or, in our case, protein domains) are unclear and two homologues (I and II) found in the same species cluster together in phylogenetic analysis relative to a homologue (III) in an outgroup species [(I, II)III], they are most likely paralogues that arose after the speciation event[25]. Conversely, if the homologues cluster as [(I, III)II], gene duplication occurred before speciation. The latter situation can be observed for the two homeoboxes found in the *DUX *family, which were duplicated before the generation of the different *DUX *paralogues.

Based on the high similarity in both gene structure and predicted homeodomain sequence compared to other homeobox genes, *DUXA *probably shares a recent common ancestor with *B*, *BL *and *C*, rather than having evolved the double-homeobox independently. Although the tree clusters DUXA HD1 and HD2 separately from the other homologues, this is most likely due to artefactual long branch attraction[26] caused by the generally higher divergence of *DUXA *from *B*, *BL *and *C *(note the long branches in Figure 6a).

Assuming our hypothesis that all four *DUX *homologues are descendants of the same ancestral *DUX *gene is correct, identifying a single-homeobox gene at the homologous locus of one of these four is informative. If the *DUX *homeobox duplication had occurred elsewhere (at a locus unlinked to *CJ057*), the syntenic *CJ057/ANXA11 *locus in non-placental mammals would be expected to either contain no *DUX*-related gene at all, or a double-homeobox gene that arrived as a paralogue from the ancestral *DUX*. We therefore propose that the single-homeobox genes in linkage with *CJ057 *and *ANXA11 *in opossum, chicken and lizard are homologues of the ancestral *sDUX *in mammals. Although we have no synteny information for wallaby and platypus *sDUX*, we have included these genes in our phylogenetic analysis due to their similar homeodomains and gene structure.

Thus, in the common ancestor of placental mammals, duplication of the homeobox in *sDUX *gave rise to the ancestral *DUX *gene, from which all other *DUX *genes are ultimately descended. Consistent with this, *sDUX *homologues cluster together at an internal node of our tree, rather than being nested within a particular branch (Figure 6b, Additional file 6).

**Figure 8:**
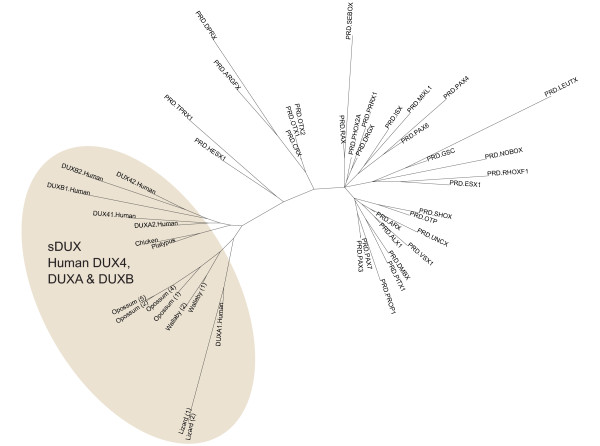
**Relationship of sDUX to known human PRD class homeodomains**. Maximum Likelihood tree based on single homeodomains. Apart from sDUX, all homeodomains are from the human orthologues (data extracted from HomeoDB[23]). Number in brackets indicates *sDUX *copy number. sDUX homeodomains cluster with DUX.

When human DUX homeodomains (DUX4, DUXA and DUXB) are compared with sDUX and human PRD class homeodomains, sDUX and all DUX proteins cluster together, further supporting their close relationship (Figure 8). Pax3 and Pax7 have been proposed to represent potential competitors for the same DNA binding sites as DUX, a hypothesis based on their similar homeodomain sequences[20,27]. However, as our tree demonstrates, Pax3/7 are not the only candidates that could be considered in this context, and indeed there are other PRD class homeodomains (e.g. HESX1, TPRX1) that could be just as or more closely related to DUX.

## Discussion

### DUX distribution across the mammalian class and divergence of homologues

The *DUX *family shows patterns of selective loss and retention of different homologues in different lineages. This stands in contrast to the distribution of another recently characterized PRD class homeobox gene, *ARGFX*, which has survived in humans but has been secondarily lost from most other mammals[28]. One of the most striking features of the *DUX *family is the loss of *Duxbl *in all examined species except rodents, with the reverse pattern for its paralogue *DUXB*.

Aside from exon shuffling and retrotransposition, gene duplication is one of the best understood mechanisms of gene evolution[29]. Following duplication, two paralogues usually meet one of two fates. If the second copy instantly confers a selective advantage ("immediate" model) or can accumulate beneficial mutations before it decays into a pseudogene ("waiting" model), it can be maintained alongside the original gene for sub- or neofunctionalization[29]. Additionally, the different genomic environment of the newly arisen paralogue may result in the use of new promoters with different expression levels or tissue specificity[30]. However, because initial functional redundancy after duplication can relax selection pressure on one of the paralogues, another likely outcome is decay of one of them[29]. This is probably what we observe in the case of *DUXB *and *Duxbl*. Which one of the two paralogues was maintained in each mammalian lineage may have been an arbitrary, stochastic decision.

Conversely, the maintenance of *DUXA *in addition to *DUXB*/*Duxbl *in many species argues for functional diversification between these gene lineages. The observed differences in gene structure and the higher homeodomain divergence in *DUXA *are consistent with this model.

For *DUXC *and *DUX4*, the evolutionary history is less clear. We cannot find any evidence for the presence of *DUXC *orthologues in rodent and primate branches, indicating that this gene may have been lost in the common ancestor of these lineages. In primates at least, the role of *DUXC *may have been filled by the intronless *DUX4*-like sequences that encode closely related homeodomains and CTD. Similarly, in the rodent lineage, mouse and rat may have a functional equivalent in *Dux*. The maintenance of intronless *DUX *genes alongside *DUXA *and *DUXB*/*Duxbl *(which also lack the conserved *DUX4 *CTD) supports separate roles for these genes.

### Implications for studying DUX4 function in model organisms

The lack of evidence for any double-homeobox *DUX *genes in non-placental mammals raises questions about some of the experimental approaches that have been employed to investigate *DUX4 *function. In a recent study, mouse *Dux *mRNA was injected into *Xenopus laevis *embryos and gastrulation defects were observed, which was suggested of being supportive of a myopathic role for D4Z4[27]. A very similar experiment using human *DUX4 *was performed by a different group[31]. In this study, observed developmental defects and increased TUNEL staining in the embryos were interpreted as evidence that in *Xenopus *there is conservation for a function of DUX4 to induce apoptosis[31].

The biological relevance of the effects of overexpressing a *DUX *homologue in an organism of a species lineage that split from the common ancestor of mammals long before the evolution of the double-homeobox gene family is unclear. One might expect that overexpression of a transcription factor in the embryo of an organism whose transcriptional networks cannot have evolved to include double-homeodomain DUX proteins in their regulation can induce cellular stress.

### Linking intronless to intron-containing DUX genes

Our data presented here and previously[1] indicate that *DUX4 *originated from retrotransposition of a processed transcript, most probably *DUXC*. As such a random integration event results in the loss of linkage with genes flanking the parental locus, there exists no syntenic relationship between intronless and intron-containing *DUX *genes. It is intriguing that of the four intron-containing *DUX *homologues, *DUXC *is the only one consistently found in tandem arrays. Although there are three adjacent copies of *Duxbl *in mouse, they are part of a much larger macroduplication that includes the neighbouring genes *Plac9 *and *Cphx*[24]. Exactly why *DUXC *and *DUX4 *(embedded in D4Z4) in particular should have repeatedly undergone duplications and maintained these copies is an unresolved question.

In rodents, the most promising candidate for a functional equivalent of *DUX4 *is *Dux*[1]. While there are fewer clues for the origin of the rodent *Dux *array than for that of *DUX4*, it probably arose from an independent retrotransposition event of an intron-containing *DUXC *homologue[1]. We base our opinion of a possible functional equivalence of Dux and DUX4 on their common, conserved C-terminal domain, which they both share with DUXC, and their shared organisation into high-copy number tandem arrays[1]. However, we note that the conserved CTD could have originally been present in all the paralogues and then lost. In that case, *Dux *could have a parental origin in a different *DUX *paralogue. Based on our trees, the origins of *DUX4 *are more likely to lie with *DUXC*, but a rapid divergence of *Duxbl *or of *DUXA *could render the homeodomain clustering of *DUX4 *with *DUXC *an artefact of our Maximum Likelihood analysis (Additional file 5 &6).

However, our data do not support the hypothesis of Wu *et al*. that mouse *Duxbl *is the orthologue of human *DUX4*, which they base on the relatively high amino acid similarity between their respective homeodomains[24]. In a separate study, either human *DUXA *or *DUX4 *were suggested as candidates for the human *Duxbl *orthologue, again based on amino acid identities[18]. Because *DUX4 *is most likely to have lost its introns through retrotransposition, it cannot be the orthologue of an intron-containing gene such as *Duxbl*[24]. Here, we have identified the human orthologue of mouse *Duxbl*, and it is human *DUXBLP *(a decaying pseudogene), located on 10q22.3. If *DUX4 *had arisen as a direct descendant of *Duxbl *by a duplication event that simultaneously resulted in the loss of all introns (in other words, retrotransposition), it could presumably be called a retrogene paralogue of *Duxbl*. It is interesting to note that after retrotransposition, *DUX4 *acquired introns in the 3' untranslated region[12,19,32]. This is rather unusual, as in most known cases of retrogenes that have acquired introns this has occurred in the 5' UTR, probably because introns distal to the stop codon usually destabilize the transcript via non-sense mediated decay (NMD) and can be selected against[33]. One example of a retrogene with an intron within the 3' UTR is *ENSG00000182814*[33]. This may represent an example of evolved NMD repression of transcript levels[33], a mechanism that should not be ruled out for *DUX4 *regulation.

Orthology and paralogy are entwined concepts defined by two fundamentally distinct evolutionary processes that give rise to the different homologues in a gene family, with important implications for gene function[34]. Orthologues are separated only by speciation events and represent the direct descendants of a gene in a common ancestor, and therefore usually perform equivalent functions in different species. Conversely, paralogues arise by gene duplication within a given species lineage, and it is an intrinsic consequence of the (initial) redundancy between the two copies that one paralogue can be released from purifying selection pressure and evolve to perform related or (crucially) novel functions[34]. So, if *DUX4 *did indeed originate from *DUXC*, its relationship to *Duxbl *can perhaps be described as it being a retrogene paralogue of a paralogue (*DUXC*) of *Duxbl*, not an orthologue. These definitions and distinctions are important if the findings of functional studies of one gene (*Duxbl*) are to be used to infer the putative functions of another gene (*DUX4*), as suggested in the study cited above[24]. Potential functional divergence between different gene family members would increase the uncertainty of such inferences. Nevertheless, these studies of rodent *Duxbl*[18,24] provide the first evidence of *in vivo *functions of any DUX protein, and are valuable contributions.

### Birth of the double-homeodomain ancestor

With *sDUX*, we have identified a putative single-homeobox homologue of *DUX *genes in non-placental animals. Based on the similarity between the sDUX and DUX homeodomains and the good synteny information (*CJ057 *and *ANXA11*), we deem it likely that in the common ancestor of the eutherian mammals a duplication of homeoboxes occurred in *sDUX*. This created the ancestral *DUX*, which ultimately gave rise to all intron-containing and intronless *DUX *genes. Our data therefore also imply that the origins of *DUXA *lie with *sDUX *and the other *DUX *family members and not with *CRX*, as suggested previously[16].

We noticed a consistent feature of our gene structure maps (Figure 5 Additional file 2, 4, 7, 8). In almost all homologues, the intron between the two sets of homeodomain encoding exons (intron 3) is comparatively small. We believe this may be a result of the duplication mechanism.

With *sDUX *present in multiple local copies in opossum, lizard and wallaby, it is easy to imagine that two closely spaced copies with identical orientations may arise. Two possible mechanisms for this are misaligned homologous recombinational repair and unequal crossing-over, which can be facilitated by repetitive elements such as SINEs[35]. Such tandem duplication *cis *events have played a major role in the evolution of other homeobox genes, for example the duplications of the *NK*-like and *Hox*/*ParaHox *genes[36].

**Figure 9:**
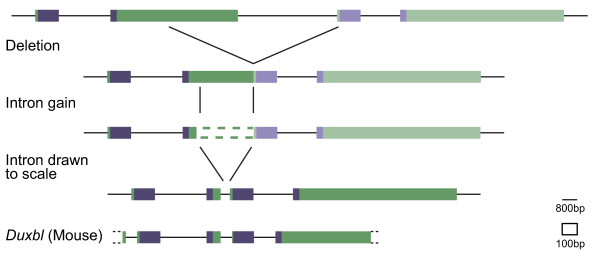
**Homeobox duplication in *DUX *genes**. Hypothetical scenario of homeobox duplication at the *sDUX *locus. As an illustrative example, the two tandem wallaby *sDUX *copies are shown with all sizes and distances to scale. A deletion could join the two genes leaving a single intact *DUX *ancestral gene. New intron redrawn at 1/8^th ^size (intron scale). Subsequent splicing changes could result in intron gain. Mouse *Duxbl *is shown for comparison.

A tandem *sDUX *scenario can indeed be observed at the wallaby locus (Figure 7c, Figure 9). Given such an arrangement, one local deletion could join the two together with a one in three chance of maintaining the reading frame. Provided the extra homeodomain at the N-terminus does not interfere with the function of the joined HD2 and its final exon, one can readily imagine natural selection to allow such a change to occur. A large open reading frame such as that in the final exon of *sDUX *(mean size of the second exon in ten identified *sDUX *copies = 989 base pairs) would allow for many possible 5' deletion breakpoints that could all result in an open reading frame. Indeed, the 3' position of the deletion breakpoint may also be variable as long as both the HD2 homeobox and no stop codon are included in the fused exon. The overall mean size of intron 3 across all *DUXA*, *DUXB*, *Duxbl*, and *DUXC *genes catalogued in our study is only 634 base pairs. We speculate that this may be the consequence of such a mutational mechanism that joined the homeoboxes (Figure 9).

A recent quantitative study analysed 330 independent events of protein domain gain and showed that gene fusion is a major mechanism, preceded by gene duplication in four out of five cases [37]. In the study sample, 71% of events occurred at protein termini and included more than one exon[37].

Our proposed model for *DUX *homeobox duplication is a simple mechanism requiring only one deletion, the size of which may be relatively variable and should allow retention of the original splice signals. A similar gene fusion by deletion has been described for the *D. melanogaster *gene *Sdic*. A tandem duplication of *Cdic *(cytoplasmic dynein intermediate chain) and *AnnX *(annexin X) followed by three local deletions joined several exons of these two genes together to create the novel gene *Sdic*[38].

Alternative molecular mechanisms for joining two neighbouring genes include readthrough transcription, which depends on mutation of the stop codon and the transcriptional termination signal in the first copy[29], and co-transcription by intergenic splicing[35]. In the latter model, pre-existing splice donor and acceptor sites from both neighbouring genes can be used to create a novel fusion gene. In such a case, a gradual erosion of the internal intron 3 could have yielded a *DUX *like gene structure.

## Conclusions

We have characterized the evolutionary history and relationships of the double-homeodomain encoding *DUX *gene family and identified a likely candidate for its common ancestor. Gene duplication followed by a fusion event most likely created the double homeobox in the last common ancestor of placental mammals as these are the only species where *DUX *genes have been found. Our study illustrates how the true underlying relationships of homologues in gene families can be obscured by secondary loss of orthologues and thus misunderstood, especially as genomic tBLASTn searches are likely to identify active genes maintained by selection, but miss decaying pseudogenes that may represent the true orthologues. Our data reinforce the view that the inference of relationships between genes should take into consideration synteny information, phylogenetic analysis of amino acid sequence evolution and consider that orthologues may no longer be present in a species. This is particularly important if, as for *DUX4*, different homologues are studied as models, especially if these studies extend to different organisms. With recently published data clearly linking *DUX4 *to FSHD pathogenesis[13], the need to understand DUX4 protein function has become more urgent. Future studies will benefit from a consideration of the evolutionary history of *DUX4*.

## Methods

### Data collection and *DUX *gene identification

Previously published data on the *DUX *gene family[1,17] was complemented and expanded by systematic interrogation of more than 30 genome databases (ENSEMBL, release May 2010, http://www.ensembl.org/Multi/blastview). Species searched included 25 placental mammals as well as opossum, platypus, chicken, lizard, frog and zebrafish (*P. troglodytes*, *G. gorilla*, *P. pygmaeus*, *M. mulatta*, *P*. *hamadryas*, *C*. *jacchus*, *M. murinus*, *T. belangeri*, *M. musculus*, *M. norvegicus*, *S. tridecemlineatus*, *C. porcellus*, *O. cuniculus*, *E. caballus*, *F. catus*, *C. familiaris*, *T. truncates*, *B. taurus*, *P. vampyrus*, *E. europaeus*, *D. novemcinctus*, *L. africana*, *M. domestica*, *O. anatinus*, *G. gallus*, *A. carolinensis*, *X. tropicalis *&*D. rerio*). Databases were surveyed for annotated *DUX *genes and searched for *DUXA*, *DUXB*, *Duxbl *and *DUXC *related sequences by whole-genome ENSEMBL tBLASTn (protein against nucleic acid) using default parameters with a near-exact matches search. For *DUXA*, *DUXB *and *Duxbl*, neighbouring anchor genes (see results section) were used to identify syntenic regions, which were then exported and searched locally for homeodomain-encoding sequences using the NCBI tBLASTn alignment tool with default parameters http://blast.ncbi.nlm.nih.gov/Blast.cgi. For *DUXC*, no suitable anchor genes could be identified due to incomplete sequence contigs flanking the *DUXC *loci and a resulting lack of linked sequences. Genes were verified as probable orthologues by considering synteny conservation, and intron-exon boundaries as well as clustering of homeodomain amino acid sequences with already characterized DUX homologues in phylogenetic trees (JalView 2.5 and PHYLIP, see below). For some genes, predicted ORFs were used in nBLAT (Ensembl) to identify any recent pseudogene retrotranspositions to aid exon identification of the intron-containing gene. If no intact orthologue (defined as an intact ORF spanning at least both homeoboxes) was found, but fragments of a decayed DUX gene could be identified in the same region (e.g. with stop codons, frameshifts, or deletion of individual exons), the orthologue was deemed to be not present or secondarily lost in that species. Where a DUX sequence was identified but the gene status (intact or pseudogene) was impossible to determine, e.g. due to local contig gaps, gene status was assigned as unclear.

### Sequence alignments and phylogenetic analysis

Predicted amino acid sequences (homeodomains were considered either concatenated or separate) were aligned using ClustalW implemented in JalView 2.5 http://www.jalview.org/[39]. JalView Average Distance or Neighbour-Joining trees based on percent identity or on a BLOSUM62 matrix were calculated initially to confirm clustering of newly identified homologues with the same class of *DUX *genes. After data collection was complete, phylogenetic analysis was performed with the PHYLIP package version 3.69 http://evolution.genetics.washington.edu/phylip.html[40]. Only homeodomain sequences from intact (and presumably functional) *DUX *homologues were included in the analysis. Datasets were bootstrapped with Seqboot (100 replicates) and unrooted Maximum Likelihood trees were calculated using Proml (Jones-Taylor-Thornton model, input order jumble = 10, non-rough analysis). Consensus trees were calculated using the majority rule option in Consense, with node bootstrap values representing the percent agreement between the 100 bootstrapped datasets. Proml analysis was then repeated on the original, non-bootstrapped dataset using the consensus tree as an input guide tree. The resulting tree with Maximum Likelihood analysis branch lengths was drawn using iTOL version 1.8.1 http://itol.embl.de/[41], and consensus bootstrap values were added manually.

### Comparative sequence analysis

The deletion breakpoint in the human, chimpanzee and orangutan *Duxbl *locus was mapped using LAGAN in mVISTA http://genome.lbl.gov/vista/mvista/submit.shtml with species specific repeat masking and default parameters[42]. To calculate dN/dS ratios, nucleic acid sequence was aligned based on translated amino acids using MEGA4 http://www.megasoftware.net/[43], and ratios were calculated using the MEGA4 implemented Nei-Gojobori method with Jukes-Cantor correction.

### Availability of sequences

All relevant sequences are included in the supplemental data (Additional file 9). Genomic loci can readily be found using the data we have provided as query sequences in ENSEMBL tBLASTn searches. Further, all human *DUX *sequences are summarized in the HomeoDB v2.0 http://homeodb.cbi.pku.edu.cn/[23].

## Authors' contributions

JEH conceived the study. AL performed database interrogation and phylogenetic analysis and drafted the manuscript. Both authors edited and approved the final version of the manuscript.

## Supplementary Material

Additional file 1 **CJ057 *alignment***. Alignment of CJ057 predicted protein. Click here for file

Additional file 2 ***DUXA *gene structures**. All labels as in Figure 1 and Figure 5. Click here for file

Additional file 3 **DUXA CTD**. Additional amino acids of extra *DUXA *exon in primates. Arrow = splice position. Click here for file

Additional file 4 ***DUXC *gene structures**. Click here for file

Additional file 5 **Concatenated HD tree with DUX4 & mDux**. Maximum Likelihood tree based on 120 amino acid concatenated homeodomains. Note the clustering of DUX4 with DUXC and the isolation of the rodent Dux node. Click here for file

Additional file 6 **Individual HD tree with DUX4 & mDux**. Tree based on 60 amino acid individual homeodomains. Click here for file

Additional file 7 ***DUXB *gene structures**. Click here for file

Additional file 8 ***Duxbl *gene structures**. Click here for file

Additional file 9 **Homeodomain amino acid sequences**. DUX homologue and homeodomain number (1 or 2) before species. Numbers in brackets after species name denote different copies. Click here for file
