# Supplementary figures and images for: A family history of DUX4: phylogenetic analysis of DUXA, B, C and Duxbl reveals the ancestral DUX gene
Source: BMC Evol Biol. 2010 Nov 26;10:364. doi: 10.1186/1471-2148-10-364 (PMC3004920; doi:10.1186/1471-2148-10-364)

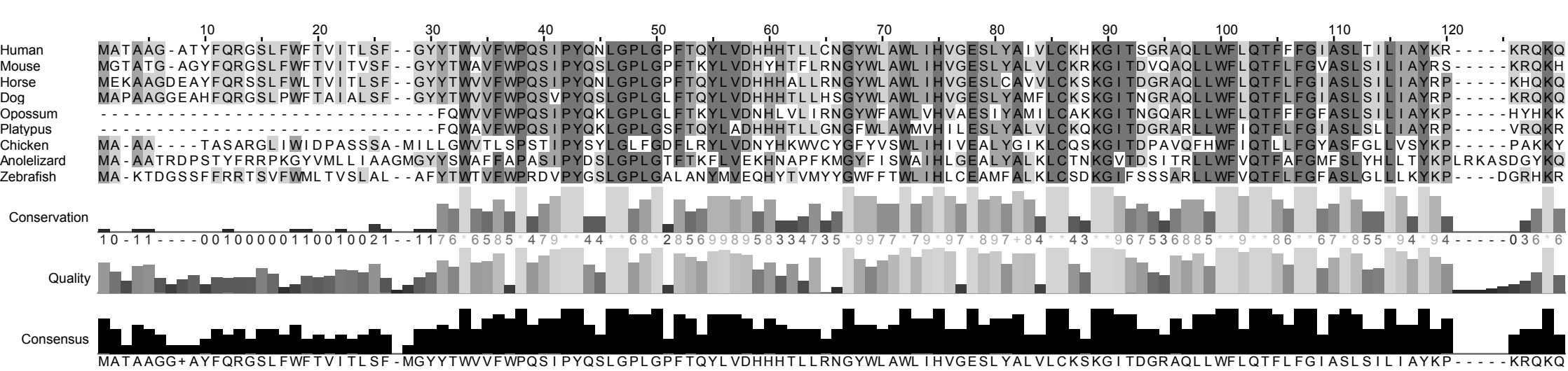

Supplement: Additional file 1 — CJ057 alignment. Alignment of CJ057 predicted protein. [file 1471-2148-10-364-S1.PDF]

# Overview of DUXA gene structure

800bp

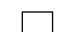

100bp

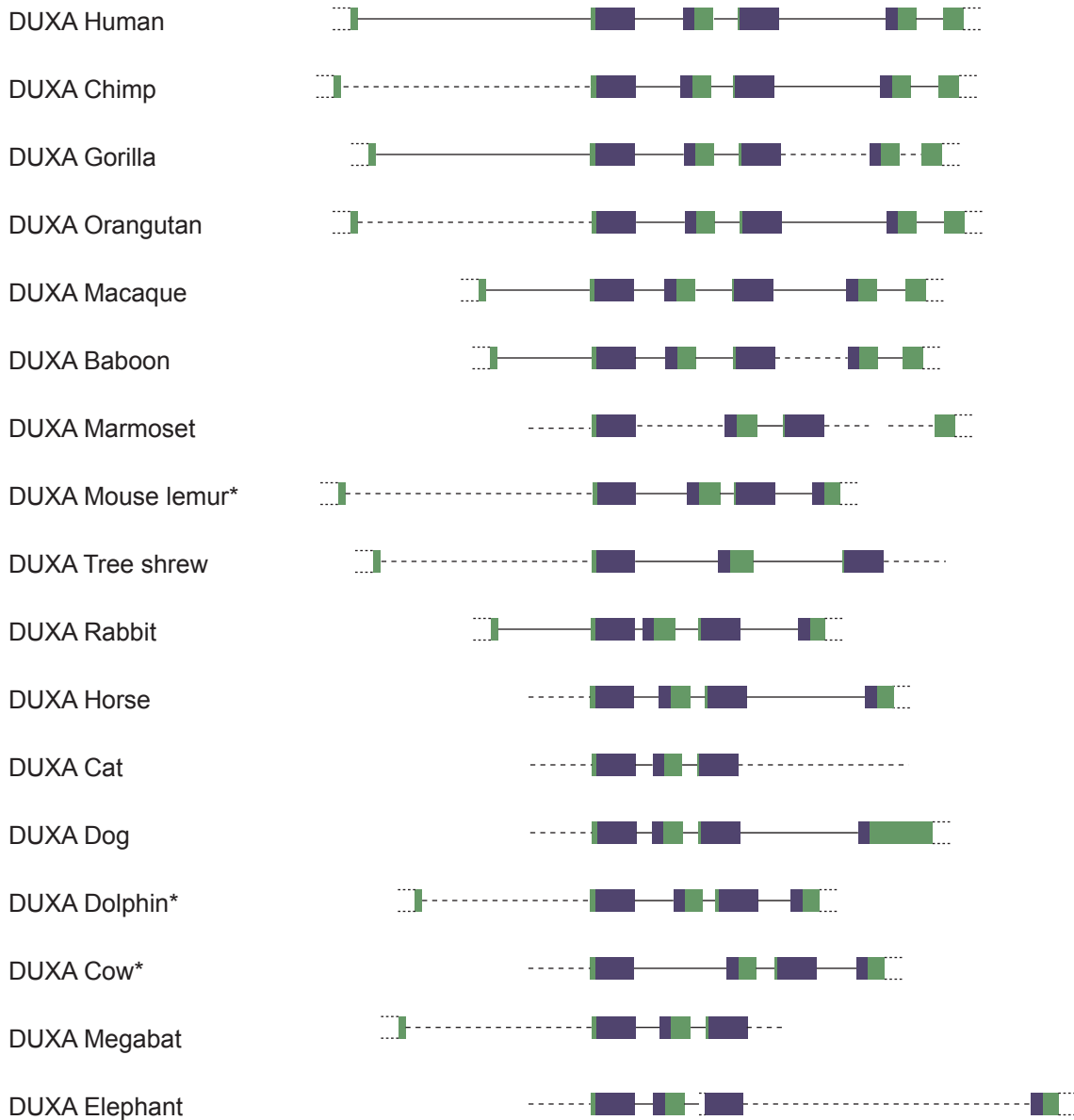

Supplement: Additional file 2 — DUXA gene structures. All labels as in Figure 1 and Figure 5. [file 1471-2148-10-364-S2.PDF]

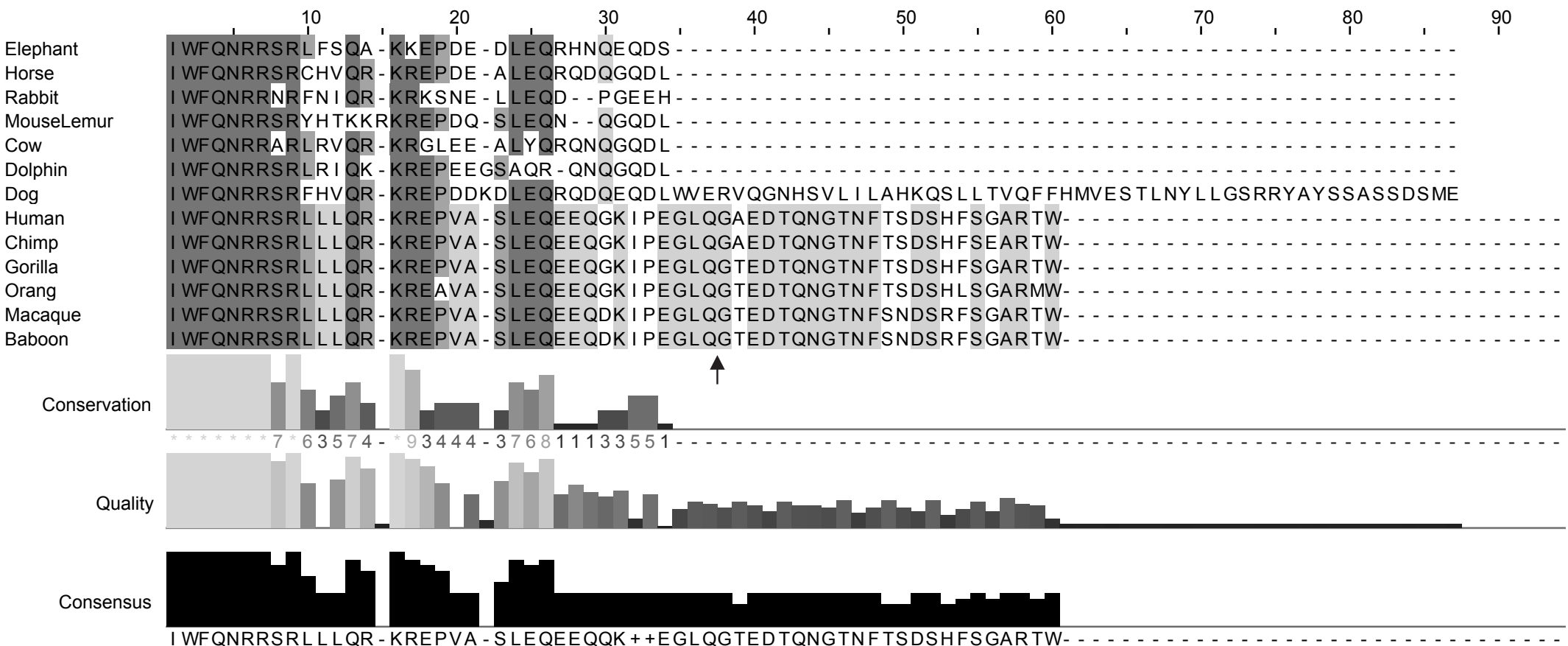

Supplement: Additional file 3 — DUXA CTD. Additional amino acids of extra DUXA exon in primates. Arrow = splice position. [file 1471-2148-10-364-S3.PDF]

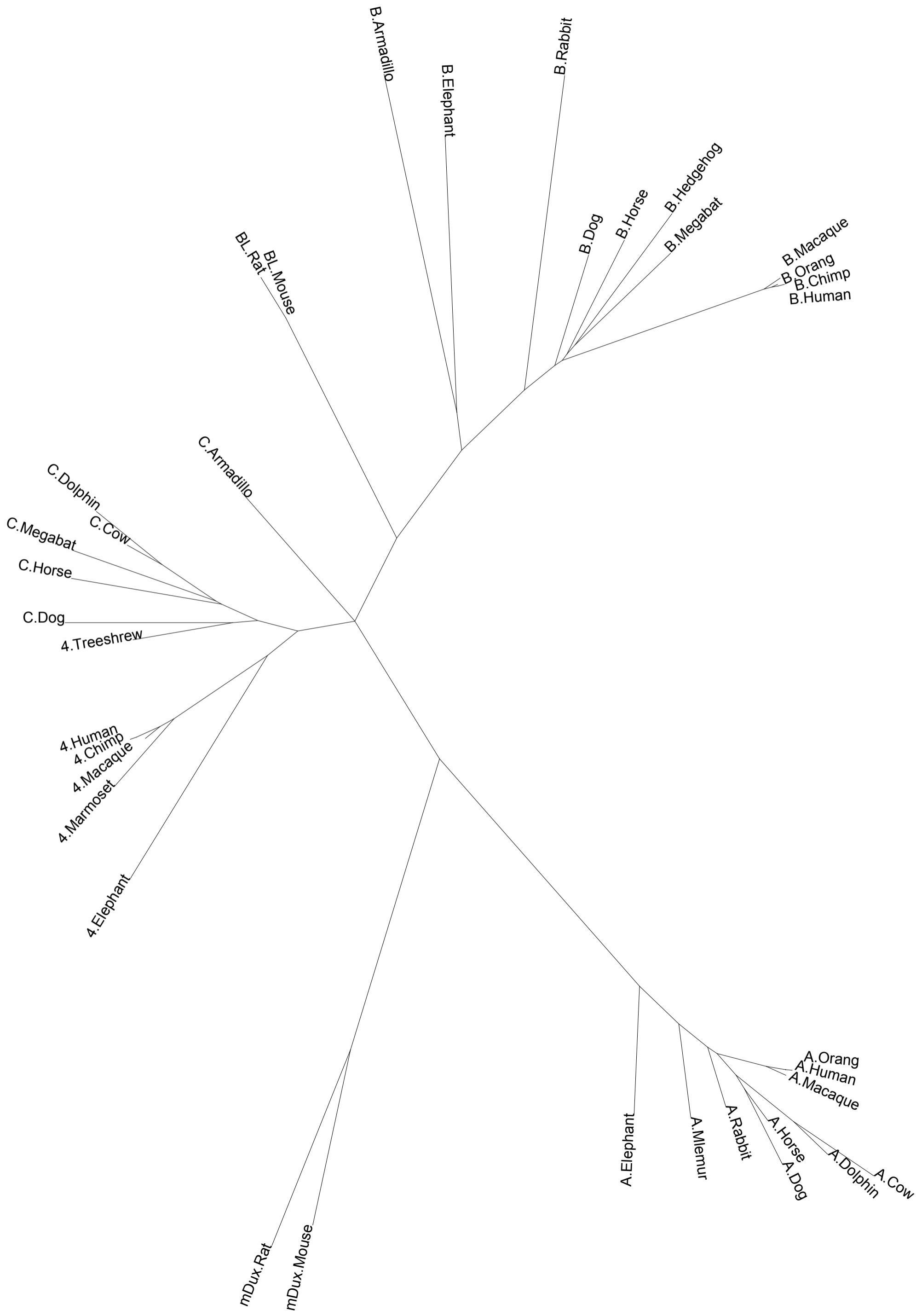

Supplement: Additional file 5 — Concatenated HD tree with DUX4 & mDux. Maximum Likelihood tree based on 120 amino acid concatenated homeodomains. Note the clustering of DUX4 with DUXC and the isolation of the rodent Dux node. [file 1471-2148-10-364-S5.PDF]

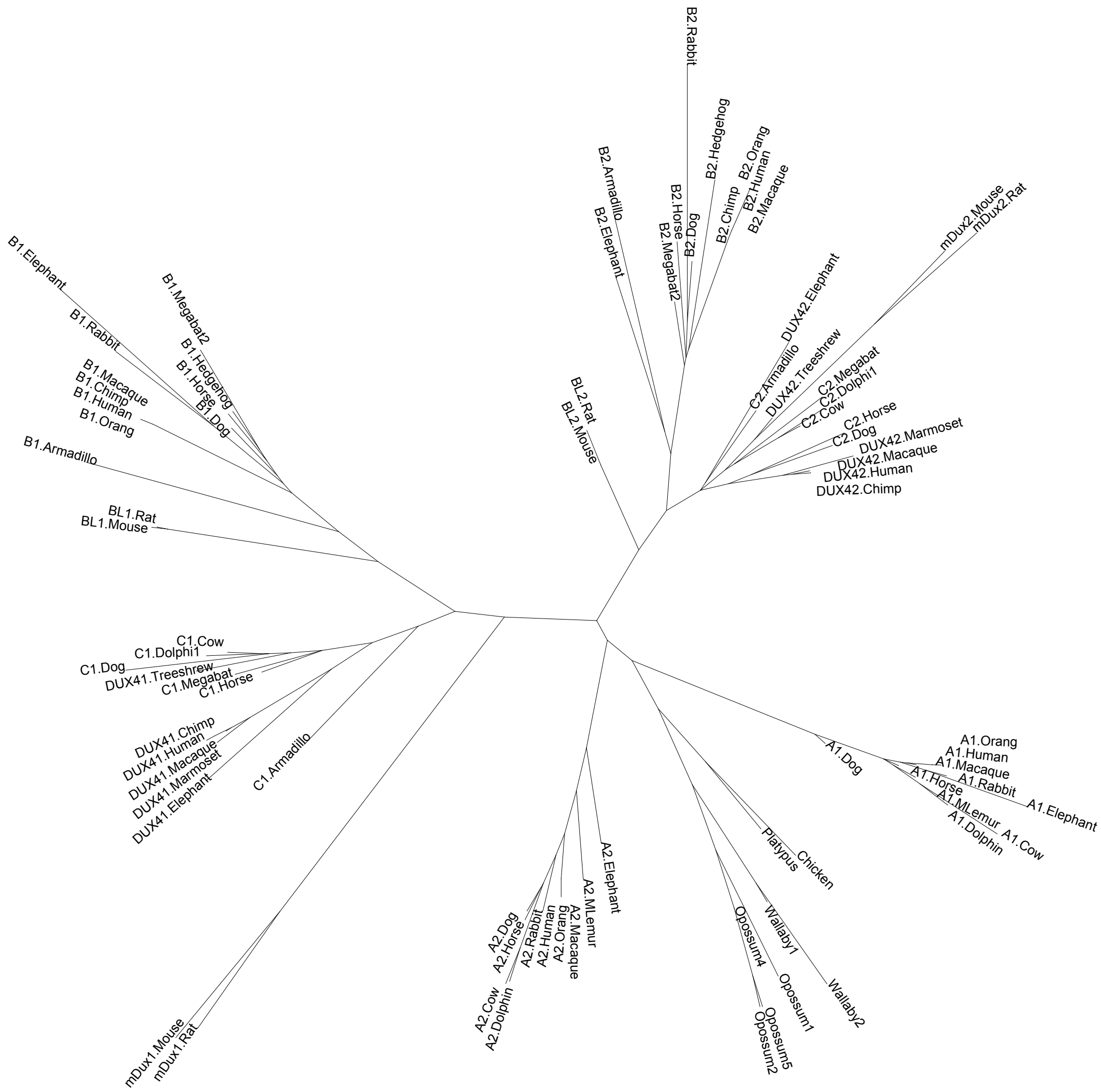

Supplement: Additional file 6 — Individual HD tree with DUX4 & mDux. Tree based on 60 amino acid individual homeodomains. [file 1471-2148-10-364-S6.PDF]

# Overview of DUXB gene structure

800bp

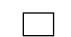

100bp

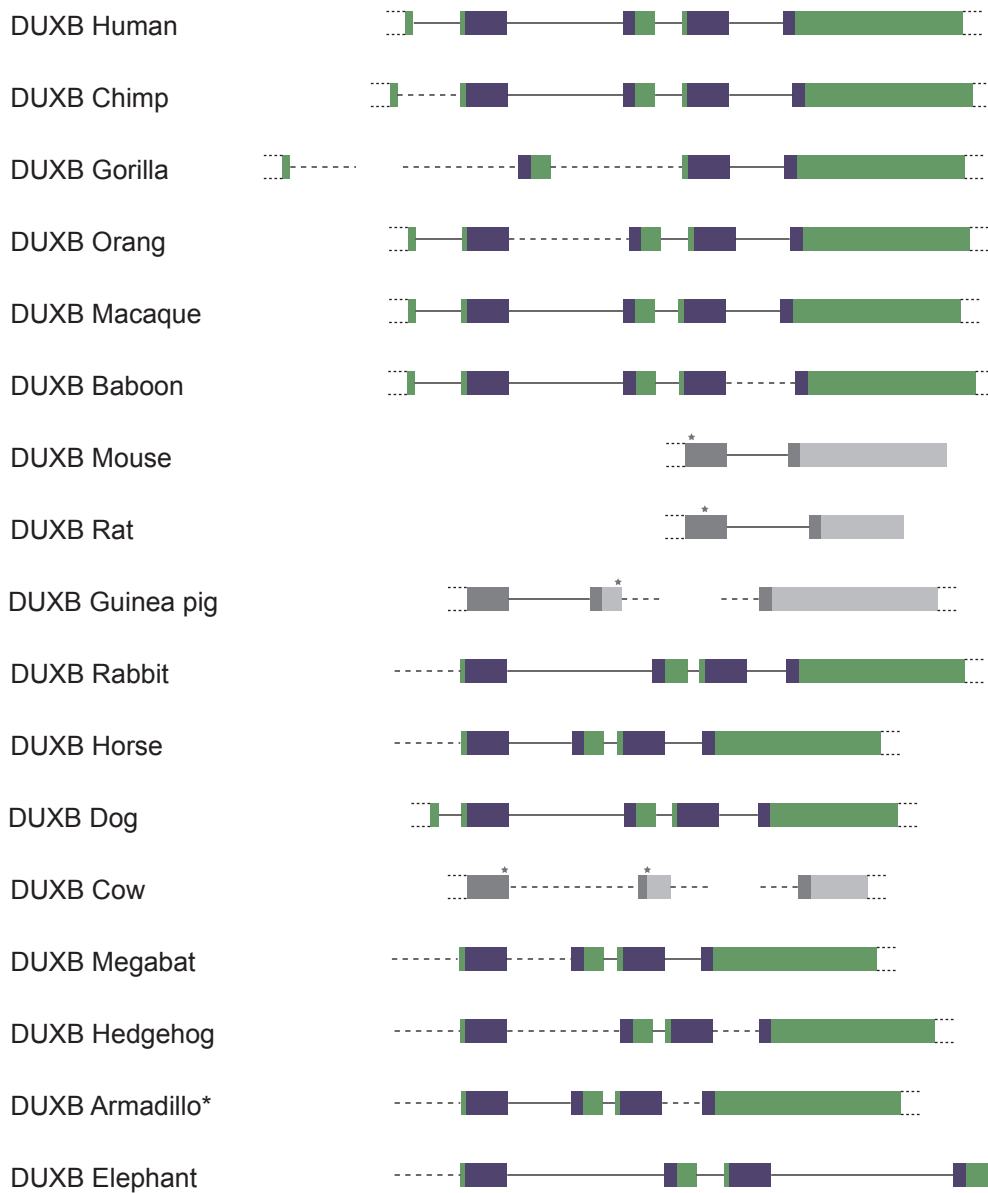

Supplement: Additional file 7 — DUXB gene structures. [file 1471-2148-10-364-S7.PDF]
