# Supplementary material for: A family history of DUX4: phylogenetic analysis of DUXA, B, C and Duxbl reveals the ancestral DUX gene
Source: BMC Evol Biol. 2010 Nov 26;10:364. doi: 10.1186/1471-2148-10-364 (PMC3004920; doi:10.1186/1471-2148-10-364)
Supplement: Additional file 4 — DUXC gene structures. [file 1471-2148-10-364-S4.PDF]

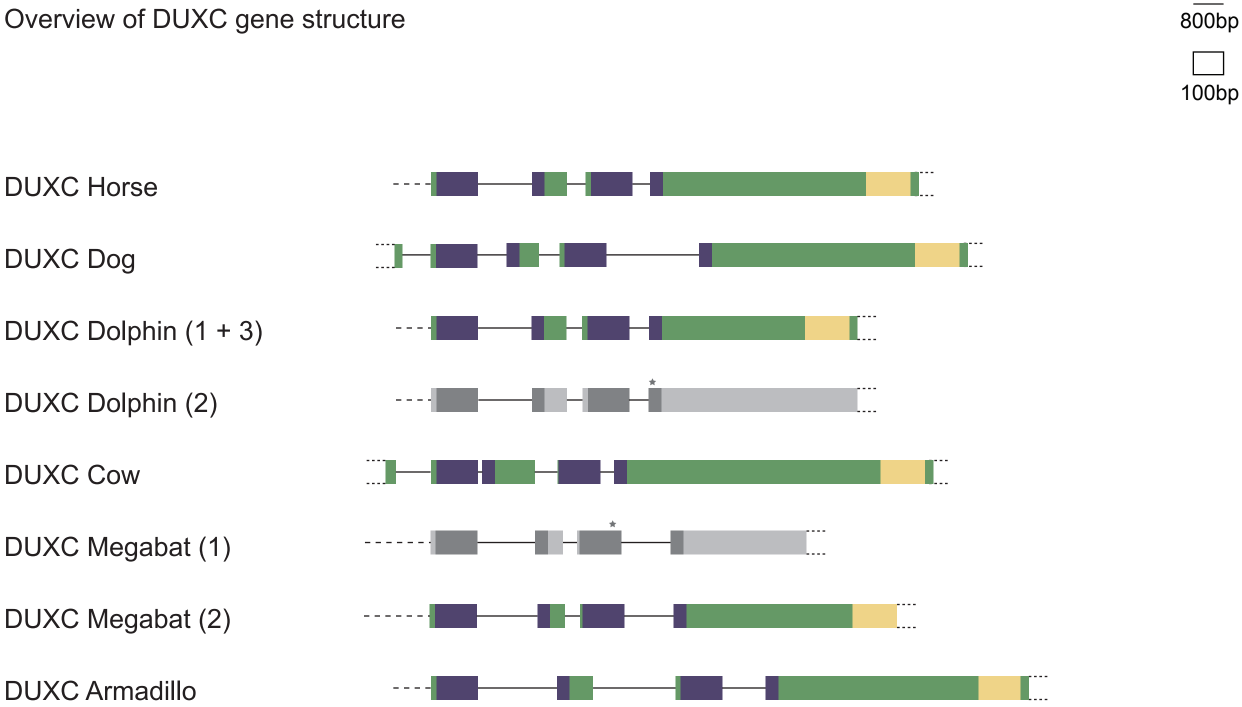
Overview of DUXC gene structure
800bp
100bp
DUXC Horse
DUXC Dog
DUXC Dolphin (1 + 3)
DUXC Dolphin (2)
DUXC Cow
DUXC Megabat (1)
DUXC Megabat (2)
DUXC Armadillo
