# Supplementary material for: A family history of DUX4: phylogenetic analysis of DUXA, B, C and Duxbl reveals the ancestral DUX gene
Source: BMC Evol Biol. 2010 Nov 26;10:364. doi: 10.1186/1471-2148-10-364 (PMC3004920; doi:10.1186/1471-2148-10-364)
Supplement: Additional file 8 — Duxbl gene structures. [file 1471-2148-10-364-S8.PDF]

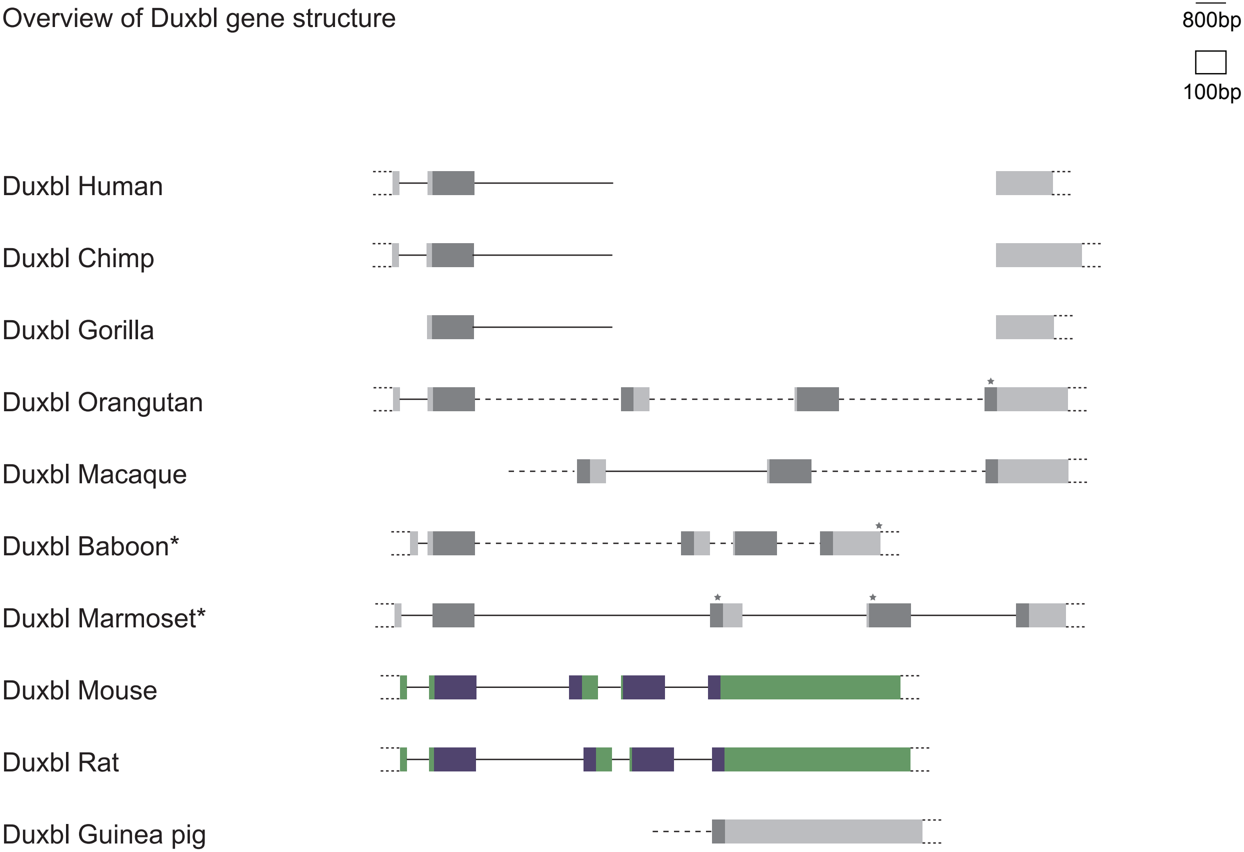
Overview of Duxbl gene structure
800bp
100bp
Duxbl Human
Duxbl Chimp
Duxbl Gorilla
Duxbl Orangutan
Duxbl Macaque
Duxbl Baboon*
Duxbl Marmoset*
Duxbl Mouse
Duxbl Rat
Duxbl Guinea pig
