# Supplementary material for: A family history of DUX4: phylogenetic analysis of DUXA, B, C and Duxbl reveals the ancestral DUX gene
Source: BMC Evol Biol. 2010 Nov 26;10:364. doi: 10.1186/1471-2148-10-364 (PMC3004920; doi:10.1186/1471-2148-10-364)
Supplement: Additional file 9 — Homeodomain amino acid sequences. DUX homologue and homeodomain number (1 or 2) before species. Numbers in brackets after species name denote different copies. [file 1471-2148-10-364-S9.PDF]

A1.Human
HRRCRTKFTEEQLKILINTFNQKPYPGYATKQKLALEINTEESRIQIWFQNRRARHGFQK
A2.Human
ARRCRTTYSASQLHTLIKAFMKNPYPGIDSREELAKEIGVPESRVQIWFQNRRSRLLLQR
A1.Orang
HRRCRTKFTEEQLKILIDTFNQKPYPGYATKQKLALEINTEESRIQIWFQNRRARHGFQK
A2.Orang
ARRCRTTYSASQLHTLIKAFMKNPYPGIDSREELAKEIGVPESRVQIWFQNRRSRLLLQR
A1.Macaque
HRRSRTKFTEDQLKILINTFNQKPYPGYATKQKLALEINTEESRIQIWFQNRRARQRFQK
A2.Macaque
ARRCRTTYSTSQLHTLIKAFVKNPYPGIDSREELAKEIGVPESRVQIWFQNRRSRLLLQR
A1.MouseLemur
CRRSRTKFTEDQLKILIHAFNQKPYPGFATKQRLASEINTEESRIQIWFQNRRARHRFQK
A2.MouseLemur
ARRSRTCYSATQLHALVQAFIKNPYPGIECREQLAKEIGVPESRVQIWFQNRRSRYHTKK
A1.Rabbit
YRRSRTRFTEEQLKILINAFNQKPYPGYATKQKLASEINTEESRIQVWFQNRRARHRFQK
A2.Rabbit
SRRCRTNYSSSQLHTLIKAFMSNPYPGIDSREQLAKEIGVPESRIQIWFQNRRNRFNIQR
A1.Horse
RRRSRTKFTEDQLKILINAFNQKPYPSYATKQRLALEINTEESRIQIWFQNRRARHRFQK
A2.Horse
DRRCRTSYTSSQLHTLIEAFMNNPYPGIDSRQQLAIEIGVPESRIQIWFQNRRSRCHVQR
A1.Dog
NRRSRTKFTEDQLKILIKAFDQNPYPGYATKQRLALEVNAEESRIQVWFQNRRARHQCQK
A2.Dog
DRRCRTSYTSSQLHTLIEAFTNNPYPGIDTREQLAKEIGIPESRVQIWFQNRRSRFHVQR
A1.Dolphin
CRRSRTKFTEEQLKILVDAFNKKPYPGYAIKQRLALEVNTEESRIQIWFQNRRARHRFLK
A2.Dolphin
DRRCRTSYTSSQLQTLMNAFMENPYPGIDSRQQLAEDIGVPESRVQIWFQNRRSRLRIQK
A1.Cow
CRRSRTSFTEEQLKILVQAFSQNPYPGYTAKQRLAVEINAEESRIQIWFQNRRARHGFLK
A2.Cow
DRRCRTSFTSSQLQTLKNAFTENPYPGIDSREQLAEEIGVPESRVQIWFQNRRARLRVQR
A1.Elephant
YRRSCTKFTGEQLKILIDTFNRKPYPNYATRQKLASEINIEEPRIQIWFQNRRARHPFRK
A2.Elephant
ARRSRTYYNCSQLHILVEAFENNPYPGIVSREQLAEEIGVPESRVQIWFQNRRSRLFSQA
B1.Human
FWRNRIQYNQSQKDILQSWFQHDPFPDKAAREQLAKEIGVPESNIQVWFKNYRVKQRKLD
B2.Human
ARQKQTFITWTQKNRLVQAFERNPFPDIATRKKLAEQTGLQESRIQMWFQKQRSLYLKKS
B1.Chimp
FWRNRIQYNQSQKDILQSWFQHDPFPDKAAREQLAKEIGVPESNIQVWFKNYRVKQRKLD
B2.Chimp
ARQKQTFITWSQKNRLVQAFERNPFPDIATRKKLAEQTGLQESRIQMWFQKQRSLYLKKS
B1.Orang
FWRNRIQYNHSQKDILQSWFQHDPFPDKAAREQLAKEIGVPESNIQVWFKNYRVKQRKLD

B2.Orang
ARQKQTFITWTQKNKLVQAFERNPFPDIATRKKLAEQTGLQESRIQMWFQKQRSLYLKKS
B1.Macaque
FWRNRIQYNQSQKDILQSWFQHDPFPDKAAREQLAKEIGVPESNIQVWFKNYRVKQRKLD
B2.Macaque
ARQKQTFITWTQKNKLVQAFERNPFPDIATRKKLTEQTGLQESRIQMWFQKQRSLYPKNC
B1.Rabbit
AFQGRIAYTENQKGILLAWFEQNPNPNKASRELLAKEIGIPASKIQTWFKNQRRKQKQLE
B2.Rabbit
AREDQFPITEAQRRILAQAFERDRFPDINTMKKLAKRVGIRASRIQMWFQKERALCSLQN
B1.Horse
TWQGRVVYSQSQKDILQKLFQHSPYPDKATREKLAKEIGIAESKIQIWFKNHRAKQRQLG
B2.Horse
ARRDQTSLTRSQSDILVGAFERKRFPDIVARKKLAKQTGIRESRIKMWYQNRRSLYPGQS
B1.Dog
TWQGRIVYNQSQKDILQEWFKQNPYPDKATRKQWAKEIGIPESEIQIWFKNHRAKQRRLE
B2.Dog
ARQDWTPITRSQSHTLVQAFERNHFPDITTRKKLAKKMGIQEPRIQVWFQNQRSLYPGQS
B1.Megabat(2)
TWKVRTVYNQSQKDILQKWFEHNPYPDTATRRQLAKEIGIPEYNIQIWFKNQRTKRRLLG
B2.Megabat(2)
ARQDQTSNTRSESNIPVQAFDRNQFPDIATRRILAKQTGIQESRIQMRFQNQRSLHPGQS
B1.Hedgehog
AWQGRIVYNQSQKDILQKWFQHNPYPDKPSREQMAKEIGIPEYKIQIWFKNHRSKQRQLG
B2.Hedgehog
VKQDQTFGRRSPSTMLVQDFERSQSPDVATRKKMSKQTSIQESRIQKWFQNGRSLYPGQS
B1.Elephant
KVQKRTAYSQRQKSILQVWFEYNPHPNKATREQLAEDIGVPEHKIQRWFRDQRKKQRQLR
B2.Elephant
ARSNQTSIMRCQTSTLLQAFQENQFPGLAATEKLAKQTEVPKSRIQMSFQNQEAQHLGHS
B1.Armadillo
CRRPRLVYSKSQKDILKAWFDKNPYPQWETRKQIAKEIGVPELKIERWFRNFRMKQRRLV
B2.Armadillo
SRRPQTSTMRSQTSTPGQAFEKNQSPDSTTRGELPKQTGFPEPRVQKWFLNQSDQHPEQS
BL1.Mouse
ARRRRIILTQSQKDTLRVWFEKNPNPDLATRGHLAKELGISESQIMTWFQKHRKIRKQAE
BL2.Mouse
ARRSRTHFTKFQTDILIEAFEKNRFPGIVTREKLAQQTGIPESRIHIWFQNRRARHPDPG
BL1.Rat
ARRRRIILNQSQKDTLRVWFEKNPNPDLATRGHLAKKLGISESQIMTWFQKHRKIRKQVE
BL2.Rat
AGRSRTHFTKFQIDILIEAFEKNRFPGIVTREKLAQETGIPESRIHIWFQNRRARHPDPK
C1.Horse
SRRRRIVLKASQRDALRAAFQQNPYPGIATRERLAQEIDIPECRVQVWFQNQRRRHLRQS
C2.Horse
GGRKRTHITPWQTGILLESFQKDRFPGIATREELARQTGIPEARIQVWFQNRRARHPDQS
C1.Dog
PRRRRLVLTASQKGALQAFFQKNPYPSITAREHLARELAISESRIQVWFQNQRTRQLRQS
C2.Dog
GRRKRTSISASQTSILLQAFEEERFPGIGMRESLARKTGLPEARIQVWFQNRRARHPGQS

C1.Dolphin(1)
SRRRRLVLSLSQKDTLQALFQQNPYPGITTREWLARELDIPESRIQVWFQNQRRRRLKQS
C2.Dolphin(1)
ARRKRTFISPSQTRVLKQAFERDRFPSIAAREELAHQTGIPEPRIQIWFQNRRARHPKQS
C1.Cow
SRRRRLVLKPSQKDALQALFQQNPYPGIATRERLARELGIDESRVQVWFQNQRRRRSKQS
C2.Cow
ARRKRTVISPSQTRILVQAFTRDRFPGIAAREELARQTGIPEPRIQIWFQNRRARHPQRS
C1.Megabat
SRRRRIVLKPHQKEALYTLFQQNPYPGITTRTQLAQEIGLPESRIQVWFQNQRRRHLKQS
C2.Megabat
ARRKRTPISPSQRCVLIQAFEKNRFPSIVTREDLARRTGIPECRIQVWFQNRRARHPGQS
C1.Armadillo
ARRKRIFLDESQKGALQALFEQNHYSSLAAREKLAKEIGIPEDRIQIWFQNRRSRCLREG
C2.Armadillo
ARRKRTRITRSQSSILVEAFEQNRFPGITTREGLATQTGLPESRIQIWFQNRRARYPGKT
Opossum(1)
KRRKRTQFTKEQISLLLCYFERNPYPGIGEREELSRMTNIEESRIQVWFQNRRARQGKGP
Opossum(2)
ARRKRTIFSKVQLDTLIDYFQKNNYPGIKEREQLSKMINVPEDRIQVWFQNRRARKSLNP
Opossum(4)
ARRKRTIFSKAQLNILINYFQKNNYPGIKEREQLSKMTNVPESRIQVWFQNRRARQGKGP
Opossum(5)
ARRKRTIFSKAQRNILIDYFQKDNYPGIEEREQLSKMINVPEDRIQVWFQNRRARRSLNP
Lizard(1)
RRRERTQFSVEQVKVLQASFNLQRYPDYERRKKLGEDIHVDEGRVAVWFQNRRARHLNTG
Lizard(2)
RRRERTQFSVEQVKALEASFNLQRYPDYERRKKLGEDIHVDEGRVAVWFQNRRARHLNTG
Wallaby(1)
SRRKRTVFTKEQLNVLINYFAKNPYPGITEREELAERLNTEESRIQVWFQNRRARQQQKW
Wallaby(2)
SRRKQTVFTNEHLNVLTSYFAKKSYPGIIEREELAERLNTEESRIQIWFQNRRARHSTQK
Platypus
ARRKRTTFNKTQLEILVKSFNKDPYPGIGVREHLASLIQIPESRIQVWFQNRRARQLGQK
Chicken
GRRKRTSFSKAQLELLVRTFEKQPYPGIALREQLSGLTDIPESRIQVWFQNRRARQLNRK
mDux1.Mouse
RRRRKTVWQAWQEQALLSTFKKKRYLSFKERKELAKRMGVSDCRIRVWFQNRRNRSGEEG
mDux2.Mouse
GRRPRTRLTSLQLRILGQAFERNPRPGFATREELARDTGLPEDTIHIWFQNRRARRRHRR
mDux1.Rat
SRRRKTVWQAWQRKALLSAFSKNKYPSFWDRQELARQIQLPSSRIRVWFQNQRSRTGEVR
mDux2.Rat
SKRPRTRLNLQQRRILVQAFERNPLPGFATREQLGQRTGLNEDTIHIWFQNRRARQARAP
DUX4.1.Human
GRPRRLVWTPSQSEALRACFERNPYPGIATRERLAQAIGIPEPRVQIWFQNERSRQLRQH
DUX4.2.Human
GRRKRTAVTGSQTALLLRAFEKDRFPGIAAREELARETGLPESRIQIWFQNRRARHPGQG
DUX4.1.Chimp
GRRRRLVWTPSQSEALRACFERNPYPGIATRERLAQAIGIPEPRVQIWFQNERSRQLRQH

DUX4.2.Chimp
GRRKRTAVTGSQTALLLRAFEKDRFPGIAAREELARETGLPESRIQIWFQNRRARHPGQG
DUX4.1.Elephant
GRRERLVLKPSQRESLRASFEQNPYPGITTREELARETGIAEDRIQTWFANRRAGLLRKS
DUX4.2.Elephant
ARRKRTAITTSQTSLLVEAFEENRYPGNEAKEELAQRTGLPRSRIHVWFQNRRARKPVQS
DUX4.1.Treeshrew
ARRRRLVLSSSQKDALQALFQQNPYPATETRERLAQEIGIPESRVQVWFQNQRTRRARQA
DUX4.2.Treeshrew
GRRKRTAISASQTRILVQAFEGNRFPGIATRERLAQQTGLPESRIHIWFQNRRARHPGRS
DUX4.1.Marmoset
GRRKRLVWTPSQRDALRASFERNPYPGMATREQLAQAIGVPEPRVQIWFQNERSRRLRQH
DUX4.2.Marmoset
GRRKRTAVTQSQTAVLLRAFERERFPDFATREELARETGLPESRIQIWFQNRRARHPGRA
DUX4.1.Macaque
GRRRRLVWTPSQREALRACFERNPYPGIATREELAQAIGIPEPRVQIWFQNERSRQLRQH
DUX4.2.Macaque
GRRKRTAVTRSQTALLLRAFQQDRFPGIATREELARETGLPESRIQIWFQNRRARHPGQG
